# Research progress and controversies in the treatment of functional constipation-related depression with probiotics and prebiotics: a narrative review

**DOI:** 10.3389/fphar.2026.1735614

**Published:** 2026-02-13

**Authors:** Qiuhua Dai, Zhongyu Duan, Chao Fang, Rong Chen

**Affiliations:** 1 College of Ethnic Medicine, Yunnan University of Chinese Medicine, Kunming, Yunnan, China; 2 First Clinical Medical College of Guizhou University of Traditional Chinese Medicine, Guiyang, China; 3 Yunnan Key Laboratory of Dai and Yi Medicines, Yunnan University of Chinese Medicine, Kunming, China

**Keywords:** depression, functional constipation, gut-brain axis, prebiotics, probiotics

## Abstract

Functional constipation (FC) commonly co-occurs with depression, with the microbiota–gut–brain axis providing a biological basis for this association. Designed as a narrative review employing systematic search strategies to ensure comprehensive coverage while accommodating evidence heterogeneity, this study evaluates the efficacy and safety of probiotics, prebiotics, and synbiotics (PP/S) in FC with comorbid depression. Using the terms “probiotic/prebiotic/synbiotic,” “functional constipation,” and “depression” as subject headings and free-text keywords, we analyzed the available evidence. Current evidence suggests that, in adults (including some older adults), PP/S are associated with improvements in spontaneous bowel movements and stool form (Bristol Stool Form Scale), alongside reductions in abdominal symptoms. Regarding psychological outcomes, depressive scores show potential small-to-moderate reductions (most consistently observed within 4–8 weeks); however, high-quality evidence specifically targeting “dual improvement” in patients with co-occurring FC and depression remains preliminary and is often limited by the high placebo response rates characteristic of functional disorders. Overall tolerability is good, with mainly mild gastrointestinal discomfort. Clinically, PP/S may serve as an additional treatment for adults with FC and depressive symptoms, especially when standard approaches are insufficient; however, given that efficacy data are largely extrapolated from primary depression studies and prone to placebo confounding, their use should be guided by the limited certainty of current evidence, given heterogeneity in strains/doses/durations/endpoints and short follow-up. More stratified, multicenter, long-term Randomized Controlled Trials (RCTs) are needed to confirm durability and identify patients most likely to benefit. Given substantial heterogeneity in strains/doses/durations and outcome definitions, we did not perform a quantitative meta-analysis or a *de novo* risk-of-bias assessment; evidence certainty ratings, where reported, are extracted from prior systematic reviews/meta-analyses.

## 1 Introduction

Functional constipation (FC) is a prevalent chronic gastrointestinal motility disorder. According to the Rome IV diagnostic criteria, FC is characterized by difficult, infrequent, or incomplete defecation (including symptoms such as straining, lumpy or hard stools, and fewer than three spontaneous bowel movements per week) persisting for at least 3 months. Crucially, FC must be clinically distinguished from Constipation-predominant Irritable Bowel Syndrome (IBS-C) and other disorders of gut-brain interaction (DGBI). While both conditions share constipation symptoms, IBS-C is defined by the predominance of abdominal pain related to defecation, whereas pain is not a dominant feature in FC. FC is clinically diagnosed primarily based on the Rome IV criteria and affects around 14% of the global population, more so in the elderly and women, and often leads to significant quality of life impairment. FC frequently co-occurs with depression, for which both symptoms and diagnosis rates are higher in FC populations; over 30% of elderly Chinese individuals with FC have significant comorbid depression or anxiety (Liang et al., 2022; Shatri et al., 2023; Hongyu et al., 2025). The two conditions also mutually exacerbate each other in outcomes: constipation can intensify depression-related symptoms, while depression can further worsen gastrointestinal function, creating a “vicious cycle” that increases healthcare resource utilization and non-satisfactory responses to laxatives (Shatri et al., 2023; Hongyu et al., 2025). The microbiota–gut–brain axis (MGBA) provides the biological foundation for this association, forming a bidirectional network of neural, endocrine, immune, and metabolic signaling. Gut microbiota dysbiosis is recognized as a common key link: patients with both constipation and depression often exhibit reduced microbial diversity, decreased short-chain fatty acids (SCFAs), lower abundance of probiotic genera (e.g., Bifidobacterium, *Lactobacillus*), and heightened inflammation (Chen et al., 2021; Liu et al., 2021; Liang et al., 2022; Irum et al., 2023; Liu X. et al., 2023; Mohan et al., 2023; Patel et al., 2025; Peng et al., 2025). Concurrently, chronic stress/depression increases intestinal permeability and induces inflammation and dysbiosis. Conversely, gut dysfunction and microbial abnormalities can influence the expression of neuromodulatory factors such as BDNF, promoting the onset and maintenance of depressive symptoms (Chen et al., 2021; Liu et al., 2021; Liang et al., 2022; Irum et al., 2023; Liu X. et al., 2023; Mohan et al., 2023; Shatri et al., 2023).

Current FC treatments primarily involve lifestyle modifications, dietary fiber, osmotic/stimulant laxatives, prokinetic agents, and biofeedback. For patients with comorbid depression, psychological intervention and antidepressant support are often necessary. Despite these available options, current standard-of-care treatments, such as osmotic laxatives and prokinetic agents, primarily target gastrointestinal motility or isolated symptoms. These approaches often fail to address the complex, bidirectional gut-brain interactions inherent in patients with comorbid depression, potentially leading to suboptimal outcomes. Furthermore, conventional psychiatric medications also fail to fundamentally improve gastrointestinal motility, with some drugs potentially exacerbating constipation, thus creating a clinical management “dilemma.” (Kang et al., 2021; Hongyu et al., 2025). Consequently, microecological interventions—probiotics, prebiotics, and synbiotics—have emerged as promising therapeutic strategies for both gastrointestinal and mood disorders (Gao et al., 2023; Musazadeh et al., 2023). Their theoretical basis lies in modulating microbiota, reducing local inflammation, and influencing MGBA-related neurotransmitters, thereby simultaneously alleviating constipation and depression (Araujo and Botelho, 2022; Radford-Smith and Anthony, 2023; Zhang Q. et al., 2023; Deng et al., 2024). Despite generally high safety profiles, existing clinical evidence remains controversial due to significant heterogeneity and uncertain efficacy, and the underlying mechanisms remain unclear (Araujo and Botelho, 2022; Radford-Smith and Anthony, 2023; Zhang Q. et al., 2023; Deng et al., 2024).

Designed as a narrative evidence synthesis covering literature published between 2015 and 2025, this review aims to address key clinical questions related to using probiotics, prebiotics, and synbiotics (PP/S) for treating functional constipation in patients with depression. The primary objectives are to: (1) evaluate the main efficacy and safety outcomes—specifically spontaneous bowel movements (SBM), complete spontaneous bowel movements (CSBM), the Bristol Stool Form Scale (BSFS), adverse events (AEs), and mood assessments including the Hamilton Depression Rating Scale (HAMD), Beck Depression Inventory (BDI), and Hospital Anxiety and Depression Scale (HADS)—of PP/S in patients with functional constipation and comorbid depressive symptoms, with a prioritization of quantitative data from adult and elderly groups while discussing pediatric studies cautiously; (2) explore the biological basis and central-peripheral signaling mechanisms of the MGBA and dysbiosis; (3) analyze how efficacy assessment metrics and patient heterogeneity affect study outcomes; and (4) compare recommendations and evidence quality across current guidelines, systematic reviews, and meta-analyses for this population (Araujo and Botelho, 2022; Radford-Smith and Anthony, 2023; Zhang Q. et al., 2023; Deng et al., 2024).

## 2 Search strategy and selection criteria

### 2.1 Methodological strengths and constraints of the narrative approach

This manuscript is structured as a narrative review to provide a broad perspective on the emerging concept of “dual efficacy” targeting the gut–brain axis. The primary strength of this approach is its flexibility, allowing for the inclusion of diverse study designs (including pilot studies and mechanistic animal trials) that are essential for exploring complex biological mechanisms but might be excluded from rigorous systematic reviews. To mitigate selection bias, we incorporated systematic search elements (e.g., specific search strings and a PRISMA flow diagram) to ensure reproducible literature retrieval.

However, this approach has limitations. Unlike a standard systematic review, we did not perform a quantitative meta-analysis or a *de novo* risk of bias assessment. This decision was made because the included studies exhibited significant heterogeneity in probiotic strains, dosing regimens, and intervention durations, which would render a quantitative synthesis statistically invalid. Therefore, the findings presented here should be interpreted as a qualitative synthesis, and any certainty of evidence ratings (e.g., GRADE) are cited directly from existing high-quality meta-analyses rather than representing an independent evaluation. Although the database search was restricted to human English-language studies, preclinical mechanistic evidence was identified via targeted hand-searching and citation chasing to contextualize MGBA pathways; these preclinical sources were not counted in the PRISMA study selection totals.

### 2.2 Search strategy

To ensure a comprehensive and reproducible review, we conducted a systematic search of PubMed, Scopus, Web of Science, and the Cochrane Library for articles published from January 2015 to October 2025. Search filters were applied to restrict results to human studies and publications in the English language. Selected seminal studies published prior to this window were included where relevant. The search strategy employed Medical Subject Headings (MeSH) terms and free-text keywords connected by Boolean operators (“Probiotics” OR “Prebiotics” OR “Synbiotics” OR “Psychobiotics” OR “*Lactobacillus*” OR “Bifidobacterium”) AND (“Functional Constipation” OR “Chronic Constipation” OR “Rome III” OR “Rome IV”) AND (“Depression” OR “Depressive Symptoms” OR “Anxiety” OR “Mood Disorders” OR “Gut-Brain Axis”).

Inclusion and Exclusion Criteria: We included randomized controlled trials (RCTs), non-randomized controlled trials, and systematic reviews involving adults, elderly individuals, or children diagnosed with functional constipation (FC) according to standard criteria (e.g., Rome III/IV). The evaluation of clinical efficacy prioritized human studies; however, preclinical (animal) models were also reviewed to elucidate the biological mechanisms underlying the microbiota–gut–brain axis interactions. Eligibility required reporting of primary gastrointestinal outcomes (e.g., SBM, BSFS) and/or psychological outcomes (e.g., HAMD, BDI), with priority given to studies assessing both domains.

**FIGURE 1:**
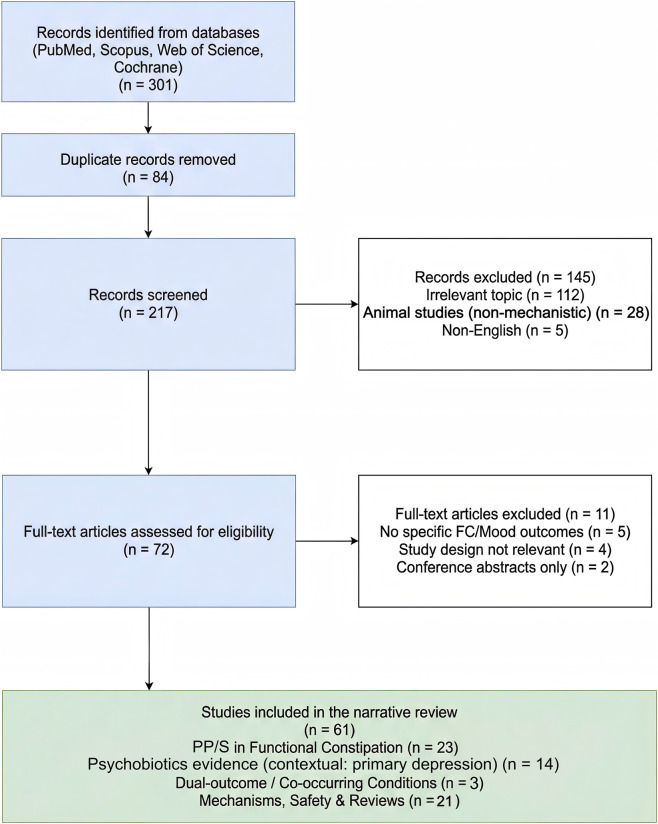
The diagram depicts the step-by-step process of identifying, screening, and including studies from four electronic databases (PubMed, Scopus, Web of Science, Cochrane Library) published between January 2015 and October 2025. Note: The PRISMA flow diagram summarizes the selection of empirical studies included in this narrative review (n = 61). Studies categorized as “psychobiotics in depression” were included to provide contextual, extrapolative background and do not represent direct evidence from FC-diagnosed cohorts. Additional methodological/regulatory sources used to contextualize clinical relevance and define MCID thresholds (e.g., U.S. Food and Drug Administration, 2012; Leucht et al., 2013; Cordaillat-Simmons et al., 2020) were not part of the study selection process and are therefore not reflected in the counts. Abbreviations: FC: functional constipation; MCID: Minimal Clinically Important Difference; PRISMA: Preferred Reporting Items for Systematic Reviews and Meta-Analyses.

We excluded duplicate publications, conference abstracts, studies primarily focusing on constipation-predominant Irritable Bowel Syndrome (IBS-C) to maintain phenotypic homogeneity, and cases of constipation secondary to organic diseases, medications, or neurological disorders. The screening process followed PRISMA principles adapted for a narrative review, as illustrated in the flow diagram in Figure 1. Finally, regarding the certainty of evidence presented in the summary tables, ratings (e.g., high, moderate, low) were adopted directly from the included systematic reviews and meta-analyses (which utilized frameworks such as GRADE or CINeMA). These ratings reflect the assessments of the original authors and do not represent a new independent formal quality assessment by the reviewers of this manuscript.

## 3 Evidence-based advances in probiotic treatment for functional constipation

To facilitate rapid comparison across populations and outcome measures, the table below summarizes key quantitative results and consistency assessments (see Table 1). Building on this summary, the subsequent Sections 3.1–3.2 provide detailed population-specific analyses to further contextualize the findings.

**TABLE 1:** Quantitative overview of primary outcomes across populations.

Outcome measure	Population	Observed clinical effect and typical magnitude	Certainty (as reported in cited meta-analyses; no *de novo* grading in this review)	References
SBM/week (spontaneous bowel movements)	Adults	Significant increase. Probiotics generally increase frequency by 0.8–1.3 SBM/week, while synbiotics may demonstrate larger effects (MD up to 4.5). Effects are most stable within 4–8 weeks	Moderate	Jayasimhan et al. (2013), Mitelmao et al. (2021), Sola et al. (2022), Deng et al. (2024), Garzon Mora and Jaramillo (2024)
BSFS (Bristol Stool Form Scale)	Adult	Improvement (softening trend) Stool consistency scores typically improve by approximately 0.3–0.6 points (shifting towards Type 3–4)	Moderate	Mitelmao et al. (2021), Deng et al. (2024), Garzon Mora and Jaramillo (2024)
Abdominal symptoms (pain, bloating)	Adults	Symptom reduction Relief rates for abdominal pain and bloating increase by approximately 15%–30% compared to control groups	Moderate	Mitelmao et al. (2021), Sola et al. (2022), Deng et al. (2024), Garzon Mora and Jaramillo (2024)
Depression scales (HAMD/BDI/HADS)	Adults	Score reduction Small-to-moderate effect sizes observed. Mean reductions typically range from 2.0 to 3.5 points, though improvements are not always synchronized with GI endpoints	Moderate	El Dib et al. (2021), Gawlik-Kotelnicka et al. (2023), Zhang Q. et al. (2023), Asad et al. (2025)
All endpoints above	Elderly	Similar trends to adults (slower response) Limited data available; efficacy trends align with adult populations but often require longer intervention periods (≥4–8 weeks) to achieve significance	Low to moderate	Sola et al. (2022), Recharla et al. (2023), Takeda et al. (2023), Ding et al. (2024)
All endpoints above	Children	Inconsistent findings Current evidence is conflicting; most systematic reviews report non-significant differences vs. placebo. Efficacy is highly dependent on diet and baseline variations	Low	Wallace et al. (2022), Dong et al. (2023), Liu L. et al. (2023), Zhang Y. et al. (2023)

SBM, Spontaneous bowel movements; BSFS, Bristol Stool Form Scale (1 = hard, 7 = watery); HAMD, Hamilton Depression Rating Scale; BDI, Beck Depression Inventory; HADS, Hospital Anxiety and Depression Scale. Certainty ratings, where shown, are extracted from the cited systematic reviews/meta-analyses (GRADE/CINeMA or equivalent frameworks). This narrative review did not perform *de novo* GRADE assessments or formal risk-of-bias grading for all included studies. This magnitude of improvement exceeds a ≥1 bowel movement/week anchor; however, CSBM-based MCID (≥1 CSBM/week) and responder rates were not consistently reported across trials, so clinical meaningfulness should be interpreted cautiously when CSBM is unavailable.

### 3.1 Conclusions from pediatric populations: lack of efficacy and developmental considerations

In distinct contrast to adult data, high-quality evidence regarding the efficacy of probiotics and prebiotics in pediatric FC remains largely negative or inconclusive. As summarized in recent systematic reviews, these interventions generally failed to demonstrate significant improvements in key outcomes such as weekly spontaneous bowel movements, stool consistency, or abdominal pain (Wallace et al., 2022; Dong et al., 2023; Liu X. et al., 2023; Zhang Y. et al., 2023; Tomsa et al., 2025). Similarly, regarding psychological outcomes, recent trials indicate that while synbiotics may aid concomitant symptom relief, their standalone efficacy in improving depressive symptoms in children is limited (Chen et al., 2024; Yang et al., 2024). Consequently, current evidence does not support the routine use of PP/S in pediatric populations. Given that the pediatric microbiome and gut–brain axis are in a state of rapid maturation, extrapolating efficacy data from adult studies to children is scientifically invalid and ethically imprudent. Clinical application in children should therefore remain restrictive and distinct from adult protocols.

### 3.2 Efficacy in adults and the elderly

Quantification of Effect: Regarding stool frequency, a recent network meta-analysis by Deng et al. (2024) synthesized data from 37 RCTs involving 3,903 patients, with 24 RCTs specifically contributing to the stool frequency outcome. The analysis confirmed significant improvements, with synbiotics demonstrating a Mean Difference (MD) of up to 4.58 bowel movements per week compared to placebo. This magnitude exceeds the ≥1 bowel movement/week clinical anchor. However, given the inconsistent reporting of CSBM across trials, strict MCID applicability remains limited and results should be interpreted with caution. In the elderly population, Recharla et al. (2023) pooled data from 5 RCTs, reporting a moderate Standardized Mean Difference (SMD) of 0.27 (95% CI: 0.05–0.50) for defecation frequency (Mitelmao et al., 2021; Sola et al., 2022; Recharla et al., 2023; Deng et al., 2024; Garzon Mora and Jaramillo, 2024). While statistically significant, this modest effect size warrants caution, as it may not consistently translate to clinically perceptible relief for all patients.

Regarding depressive symptoms, reductions in HAMD scores across various trials typically ranged from 2 to 3.5 points. Crucially, many of these reductions hover around or fall below the MCID threshold for depression (≥3 points). This suggests that while statistical significance is frequently achieved, the clinical meaningfulness of these changes often remains borderline. Furthermore, few studies explicitly reported MCID-based responder rates, which limits the interpretation of clinical relevance. Therefore, statistically significant differences should not be assumed to represent clinically meaningful benefits when MCID thresholds are not met. Nevertheless, some evidence suggests that PP/S may offer a safer long-term alternative or adjunct to traditional laxatives, particularly for maintenance therapy (Nikolova et al., 2021; Mitelmao et al., 2022; Harris et al., 2025).

Comparative Efficacy of Formulations: Evidence regarding the superiority of complex formulations is nuanced. Synbiotics exhibit robust evidence for enhanced efficacy; for instance, a specific synbiotic mixture (“Mix7”) ranked first in the network meta-analysis with a SUCRA (surface under the cumulative ranking curve) probability of 94.8%. However, the advantage of multi-strain probiotics over single strains is less definitive and appears population-dependent. In elderly subjects, single-strain interventions actually demonstrated a significant effect size (SMD = 0.49) compared to non-significant results for multi-strain formulations (SMD = 0.48), suggesting that strain specificity may outweigh strain diversity in certain cohorts (Sola et al., 2022; Recharla et al., 2023; Deng et al., 2024). This aligns with mechanistic data indicating that therapeutic effects—particularly for mood regulation—are highly dependent on specific bacterial metabolites and neuroactive compounds (Jach et al., 2023; Li et al., 2023; Rahmannia et al., 2024).

### 3.3 Influencing factors

Multi-strain versus single-strain: In adult populations, multi-strain or synbiotic PP/S formulations generally confer greater benefits in improving stool frequency and consistency (Sola et al., 2022; Deng et al., 2024).

Intervention duration: A period of ≥4–8 weeks produces more consistent effects in adult cohorts. Elderly individuals tend to respond more slowly to interventions and may require careful adjustment of both dosage and duration to achieve beneficial outcomes (Mitelmao et al., 2021; Sola et al., 2022; Deng et al., 2024).

Expanding on the significance of intervention duration, research heterogeneity further influenced results. Studies differed in baseline severity of constipation, varying lengths of follow-up, inconsistent methods for outcome measurement, and differences in blinding practices. Due to these factors, overall effects were uncertain, especially in pediatric studies (Wallace et al., 2022; Dong et al., 2023; Liu X. et al., 2023; Zhang Y. et al., 2023; Garzon Mora and Jaramillo, 2024).

Heterogeneity in product types (multi-strain vs. single-strain), dosing regimens, study designs, and outcome assessment standards remains the principal barrier. This diversity makes it difficult to establish consistent evidence-based recommendations for probiotic interventions (Sola et al., 2022; Liu X. et al., 2023; Deng et al., 2024; Garzon Mora and Jaramillo, 2024).

## 4 Research focus and clinical evidence on prebiotics for constipation

### 4.1 Primary prebiotic categories and mechanisms of action

Prebiotics are non-digestible substances that help beneficial bacteria grow and offer health benefits. Main types include Fructo-Oligosaccharides (FOS), inulin, Galacto-Oligosaccharides (GOS), and resistant starch (Erhardt et al., 2023; He et al., 2025). Building on this, these substrates are fermented by colonic microbiota (e.g., Bifidobacterium, *Lactobacillus*) to produce SCFAs, which lower colonic pH, promote intestinal motility and barrier function, and may improve constipation-related symptoms via the enteric neuroendocrine pathway (Deng et al., 2024; Erhardt et al., 2024).

### 4.2 Key clinical evidence

A summary of key evidence regarding prebiotic interventions, including specific findings on inulin and fructo-oligosaccharides (FOS) from recent randomized controlled trials and meta-analyses, is presented in Table 2. Specifically, a double-blind randomized controlled trial in adults with chronic constipation demonstrated that inulin supplementation increased weekly bowel movement frequency and improved clinical symptoms (Terren Lora et al., 2024). In addition to the RCT findings, a network meta-analysis indicated that prebiotics overall outperformed placebo, though effect sizes showed heterogeneity, with no significant differences between types (GOS/FOS/inulin) (Deng et al., 2024). Specific data on galacto-oligosaccharides (GOS) also confirm their safety and bifidogenic potential in managing constipation-related symptoms (Lee et al., 2024). Regarding safety, the profile is favorable: adverse reactions primarily include mild bloating, borborygmi, and abdominal pain, with no serious adverse events reported (Terren Lora et al., 2024).

**TABLE 2:** Summary of key evidence and findings from major studies on prebiotic intervention for functional constipation.

Study design	Population/Setting	Intervention (type and duration)	Primary outcomes	Key findings	Safety/Adverse event	References
RCT (double-blind)	Adults with chronic/functional constipation	Inulin (duration: 2–8 weeks)	Weekly bowel movement frequency; stool consistency; QoL scores	Increased frequency and softening Supplementation significantly increased weekly bowel movements and improved stool consistency compared to placebo	Mild bloating, borborygmi, and abdominal pain; No serious AEs reported	Araujo and Botelho (2022), Terren Lora et al. (2024)
Network meta-analysis	Adults (pooled data from multiple RCTs)	Multiple prebiotics (GOS/FOS/inulin) (duration: 1–12 weeks)	SBM frequency; stool consistency; microbial diversity	Superiority over placebo Prebiotics overall outperformed placebo, though effect sizes showed high heterogeneity. No significant efficacy differences were found between GOS, FOS, and inulin	Safety data were not uniformly reported across all included studies	Deng et al. (2024)
Animal Study	Mice (Loperamide-induced constipation model)	Prebiotic-containing formula (e.g., sesame candy) (duration: 10 days)	Intestinal transit rate; fecal water content	Accelerated transit Intervention reversed drug-induced constipation by accelerating intestinal transit and increasing fecal water content	Not reported in this model	Xia et al. (2022)
Mechanistic Review	General/preclinical Basis	Fermentable substrates (FOS, GOS, resistant Starch) → SCFAs	Colonic pH; motility; barrier function	Mechanism support Fermentation produces SCFAs, lowers colonic pH, and promotes motility. These physiological changes support the clinical rationale for use in FC.	N/A	Erhardt et al. (2023), Erhardt et al. (2024), He et al. (2025)
Subgroup/narrative review	Elderly/high-risk (polypharmacy, metabolic issues)	Multi-type prebiotics multi-type prebiotics vs. dietary interventions	Short-term vs. Long-term	Symptom relief; bowel regularity	Inconsistent evidence Data for complex subgroups are limited and heterogeneous; effects are less consistent than in healthy adult cohorts	Araujo and Botelho (2022), Erhardt et al. (2023), Deng et al. (2024)

RCT, Randomized Controlled Trial; GOS, Galacto-oligosaccharides; FOS, Fructo-oligosaccharides; SBM, Spontaneous Bowel Movements; QoL, Quality of Life; SCFAs, Short-chain Fatty Acids; AEs, Adverse Events; FC, Functional constipation. MCID interpretation (constipation outcomes): When CSBM is reported, an increase of ≥1 CSBM/week is commonly used as the MCID for clinical relevance; when only SBM/overall stool frequency (or symptom scales) is reported and CSBM, is not available, CSBM-based MCID cannot be directly determined and clinical meaningfulness should be interpreted cautiously.

However, age-related differences in response kinetics are notable. While adults often respond within 4 weeks, elderly individuals typically require a longer intervention duration of at least 4–8 weeks to achieve statistically significant improvements. Regarding formulation, emerging evidence suggests that multi-strain probiotics or synbiotics (combinations of probiotics and prebiotics) may exhibit enhanced efficacy compared to single-strain preparations, likely due to synergistic effects on the gut microbiota, although head-to-head comparisons remain limited.

### 4.3 Clinical evidence in co-occurring functional constipation and depression

#### 4.3.1 Evidence from dual-targeted trials

Direct evidence evaluating the “dual efficacy” of probiotics or synbiotics specifically in patients who meet diagnostic criteria for both functional constipation and mood disorders remains limited. Observational studies have firmly established the high prevalence of this comorbidity and its association with specific gut microbiota dysbiosis (Liang et al., 2022) and inflammatory markers (Shatri et al., 2023). However, interventional data addressing these targets is scarce. As summarized in Table 3, only a few high-quality RCTs have specifically targeted this comorbidity. For instance, (Zhang X. et al., 2021), reported significant improvements in constipation symptoms (e.g., stool consistency) with Lacticaseibacillus paracasei Shirota. However, critically, the reduction in depressive scores in the intervention group did not differ significantly from the placebo group, likely due to a strong placebo response common in functional gastrointestinal disorders. This suggests that while the biological link is evident in observational data, the simultaneous therapeutic benefit in clinical trials is not yet definitively established.

**TABLE 3:** Summary of studies assessing both functional constipation and depressive/psychological outcomes.

Study (author, year)	Population and sample size	Diagnostic criteria	Intervention/Observation	FC outcomes (primary)	Psychological outcomes (secondary/dual)	Key findings on comorbidity
(Zhang X. et al., 2021) (RCT)	Adults with Depression and Constipation (n = 82)	Rome IV (FC); ICD-10 (depression)	Lacticaseibacillus paracasei Shirota (LcS) vs. Placebo for 9 weeks	PAC-SYM scores; SBM frequency	HAMD-24; BDI scores	LcS significantly improved specific constipation symptoms (e.g., stool consistency). Both groups showed reduced depression scores, but no significant difference was found between LcS and placebo
(Shatri et al., 2023) (observational)	Adults with chronic functional constipation (n = 73)	Rome IV	Cross-sectional analysis of inflammation biomarkers	N/A (observational)	BDI-II scores; NLR and PLR (inflammation)	High prevalence of depression (73.0%) in FC patients. Inflammatory markers (NLR, PLR) were elevated in depressive patients but did not reach statistical significance (p > 0.05)
(Liang et al., 2022) (observational)	Elderly Chinese with FC (n = 198)	Rome IV	16S rRNA sequencing correlation analysis	SBM; BSFS	PHQ-9 (depression); GAD-7 (anxiety)	Mood disorders were prevalent (30.3% Depression). Specific gut taxa (e.g., *Bacteroides*, Aquicella) were significantly correlated with both constipation severity and anxiety/depression scores

Abbreviations: PAC-SYM, patient assessment of constipation-symptoms; SBM, spontaneous bowel movements; BSFS, Bristol Stool Form Scale; HAMD, Hamilton Depression Rating Scale; BDI, Beck Depression Inventory; PHQ-9, Patient Health Questionnaire-9; GAD-7, Generalized Anxiety Disorder-7; NLR, Neutrophil-to-Lymphocyte Ratio; PLR, Platelet-to-Lymphocyte Ratio. FC, Functional constipation. MCID interpretation (dual outcomes): For constipation, the MCID is ≥ 1 CSBM/week when CSBM is reported; for depressive symptoms, a ≥3-point reduction in HAMD is used as the MCID. If CSBM/HAMD are not reported (e.g., only SBM, PAC-SYM, BDI or exploratory mood outcomes), MCID-based clinical meaningfulness cannot be formally judged, and statistically significant changes may not necessarily be clinically meaningful.

Statistical Clarifications: Zhang X. et al. (2021): While specific symptoms (e.g., stool consistency) improved significantly, the difference in the total PAC-SYM, score between the probiotic and placebo groups was not statistically significant. Depressive scores decreased in both groups, suggesting a potential placebo effect. Shatri et al. (2023): Although inflammatory markers (NLR, PLR) were higher in patients with depression, the difference did not reach statistical significance (p > 0.05).

#### 4.3.2 Extrapolated evidence from primary psychiatric studies

While our systematic search for FC-focused clinical efficacy prioritized studies with a functional constipation diagnosis, we also summarized selected psychobiotic evidence in primary depression as contextual, extrapolative background; these studies do not constitute direct evidence from FC-diagnosed cohorts. Drawing upon established systematic reviews and meta-analyses in the field of psychiatry [e.g., (El Dib et al., 2021; Musazadeh et al., 2023)], we note that probiotic interventions in patients with primary depression often yield statistically significant improvements. However, these findings constitute extrapolated evidence when applied to the FC population. The discrepancy between the positive outcomes in these general psychiatric reviews and the mixed results in our specific FC cohort (Section 4.3.1) highlights that the pathophysiology of “FC-associated depression” (often secondary to somatic distress) may differ from primary Major Depressive Disorder, and thus findings cannot be automatically generalized without further validation.

## 5 Depression outcomes and mechanisms

### 5.1 Mechanisms of action: insights from preclinical models

The therapeutic potential of probiotics and prebiotics is largely attributed to their modulation of the microbiota-gut-brain axis (MGBA). Preclinical studies have elucidated several key pathways. First, the serotonergic pathway plays a critical role; probiotics may enhance the intestinal availability of serotonin (5-HT), a neurotransmitter essential for mood regulation, thereby improving signaling along the gut-brain axis (Rajanala et al., 2021; Suda and Matsuda, 2022; Radford-Smith and Anthony, 2023).

Second, interventions have been observed to upregulate neurotrophic factors, specifically Brain-Derived Neurotrophic Factor (BDNF) and Gamma-Aminobutyric Acid (GABA). These changes support neuroplasticity and help suppress the neuroinflammation often associated with depressive behaviors in animal models (Rajanala et al., 2021; Radford-Smith and Anthony, 2023; Patel et al., 2025).

Additionally, the fermentation of prebiotics by colonic microbiota produces Short-Chain Fatty Acids (SCFAs), such as acetate, propionate, and butyrate. SCFAs serve to lower colonic pH and enhance intestinal barrier integrity (Liu et al., 2021; Zhang S. et al., 2021; Zou et al., 2024).

**FIGURE 2:**
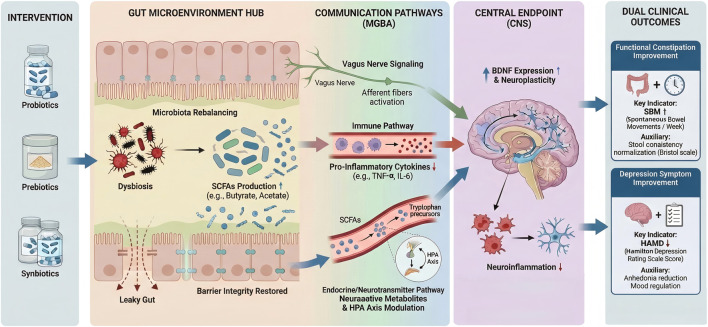
Proposed mechanisms by which probiotics, prebiotics, and synbiotics (PP/S) may improve functional constipation (FC) and co-occurring depressive symptoms via the microbiota–gut–brain axis (MGBA). Oral PP/S may modulate the gut microenvironment by rebalancing microbiota composition (e.g., increased *Bifidobacterium and Lactobacillus*), promoting short-chain fatty acid (SCFA) production, and improving intestinal barrier integrity. These peripheral changes are proposed to influence the central nervous system (CNS) through three MGBA routes: (1) neural signaling (vagus nerve/afferent activation); (2) immune signaling (reduced pro-inflammatory cytokines such as IL-6 and TNF-α); and (3) endocrine/metabolic signaling (neuroactive metabolites and HPA-axis modulation). In the CNS, these signals may reduce neuroinflammation and support neuroplasticity (e.g., increased BDNF expression). Clinically, PP/S may be associated with improved bowel outcomes (e.g., increased SBM [±CSBM] and improved stool consistency, Bristol scale/BSFS) and reduced depressive symptom severity (e.g., lower HAMD or other validated depression scales). Note on Evidence Sources: Clinical outcomes in the right panel (SBM, BSFS, HAMD) summarize findings derived from human clinical trials. The molecular and cellular mechanisms illustrated in the central pathways (e.g., vagus nerve activation, specific neuroinflammatory signaling, BDNF-related pathways) are synthesized predominantly from preclinical evidence (animal and/or *in vitro* studies) and therefore await validation in human populations. Evidence labels [Human] = supported by clinical studies; [Preclinical] = supported mainly by animal/*in vitro* studies; [Hypothesized] = biologically plausible but not yet confirmed in humans. Key: ↑ increase/upregulation/improvement; ↓ decrease/downregulation/symptom reduction; “+” beneficial clinical change. Abbreviations: BDNF: brain-derived neurotrophic factor; BSFS: Bristol Stool Form Scale; CNS: central nervous system; CSBM: complete spontaneous bowel movements; FC: functional constipation; HAMD: Hamilton Depression Rating Scale; HPA: hypothalamic–pituitary–adrenal; IL: interleukin; MGBA: microbiota–gut–brain axis; PP/S: probiotics, prebiotics, and synbiotics; SBM: spontaneous bowel movements; SCFAs: short-chain fatty acids; TNF-α: tumor necrosis factor-alpha; 5-HT: 5-hydroxytryptamine (serotonin).

This barrier restoration is crucial for preventing the translocation of bacterial toxins, thereby reducing systemic inflammation markers (e.g., IL-6, TNF-α) (Yang et al., 2022; Mohan et al., 2023; Radford-Smith and Anthony, 2023), that can negatively impact the central nervous system (Zhang X. et al., 2021; Jankiewicz et al., 2023; Zhang Q. et al., 2023; Zou et al., 2024). A schematic overview of the proposed microbiota–gut–brain mechanisms is provided in Figure 2.

### 5.2 Clinical biomarkers and physiological responses

Translating mechanistic findings to human subjects has yielded consistent but variable signals. In patients with functional constipation and comorbid depression, baseline assessments frequently reveal gut dysbiosis characterized by reduced diversity and lower levels of fecal SCFAs (Liu et al., 2021; Zhang S. et al., 2021; Zou et al., 2024).

Following biotic interventions, clinical studies have reported increases in SCFA levels and improvements in microbial diversity, which correlate with symptom relief. Furthermore, reductions in pro-inflammatory cytokines (IL-1β, IL-6, CRP) (Yang et al., 2022; Mohan et al., 2023; Zou et al., 2024), and increases in serum BDNF levels have been documented (Zhang Q. et al., 2023; Zou et al., 2024), supporting the hypothesis that these interventions exert anti-inflammatory and neuroprotective effects in humans (Rajanala et al., 2021; Suda and Matsuda, 2022; Radford-Smith and Anthony, 2023). However, the linear correlation between these biomarker changes and clinical symptom improvement remains complex and non-linear (Liu et al., 2021; Zhang S. et al., 2021; Zou et al., 2024).

### 5.3 Impact on depression scores

Regarding psychological outcomes, results remain heterogeneous. While pooled data from randomized controlled trials and systematic reviews generally suggest that probiotics and prebiotics may help alleviate depressive symptoms, demonstrating statistically significant improvements with small-to-moderate effect sizes (SMD) rather than uniform point reductions across different scales (El Dib et al., 2021; Gawlik-Kotelnicka et al., 2023; Zhang Q. et al., 2023; Asad et al., 2025). However, individual high-quality RCTs specifically targeting FC-related depression often report mixed results, with some trials observing non-significant differences compared to placebo. These benefits appear most stable when the intervention duration ranges from 4 to 8 weeks. Nevertheless, evidence regarding the long-term sustainability of these mood improvements beyond 6 months is currently limited and requires further validation (Dong et al., 2023; Radford-Smith and Anthony, 2023; Zhang Q. et al., 2023; Zhang Y. et al., 2023; Soderberg Veiback et al., 2025).

### 5.4 Translational perspective: mapping mechanisms to clinical outcomes

To bridge the gap between preclinical insights and clinical realities, it is critical to distinguish established associations from speculative pathways. Currently, the strongest human-level evidence links SCFAs—particularly butyrate—to gastrointestinal endpoints; increased fecal butyrate correlates reliably with reduced colonic transit time and improved stool consistency (BSFS) via G-protein-coupled receptor activation (Jach et al., 2023; Rahmannia et al., 2024). Conversely, the translational validity of neurotrophic and inflammatory markers is less robust. While BDNF upregulation is a consistent driver of antidepressant effects in rodent models, evidence in human trials is conflicting, with serum BDNF levels often failing to mirror clinical mood recovery (Li et al., 2023). Similarly, while reductions in pro-inflammatory cytokines (e.g., IL-6, TNF-α) theoretically underpin the “psychobiotic” effect, distinct correlations between cytokine profiles and HAMD score reductions in FC patients remain inconsistent (Zhang X. et al., 2021). Thus, while metabolic modulation (SCFAs) is a proximal driver of GI relief, neuro-immune pathways (BDNF, cytokines) currently represent plausible but largely inferred mechanisms for psychological benefits in this specific comorbidity.

## 6 Safety, contraindications, and regulatory considerations

### 6.1 Safety profile and adverse events

Overall, probiotics and prebiotics are considered safe for the general population, with the most common adverse events being mild, transient gastrointestinal symptoms such as bloating, flatulence, and abdominal distension (see Table 4). However, serious adverse events, though rare, have been documented. Case reports of bacteremia, fungemia, and sepsis have been linked to probiotic use in high-risk groups, including severely immunocompromised patients, those with central venous catheters, and critically ill individuals with impaired intestinal barrier integrity (Mayer et al., 2023; Sada et al., 2024; Xu et al., 2025). Therefore, caution is warranted when prescribing PP/S to these vulnerable populations.

**TABLE 4:** Overall tolerability and adverse events in studies of probiotics/prebiotics.

Intervention category	Common adverse events (AEs)	Observed safety profile (vs. Placebo)	Key compliance considerations	References
Probiotics	Mild gastrointestinal discomfort (abdominal distension, belching, flatulence)	No significant difference Meta-analyses indicate incidence rates are statistically comparable to placebo. Events are generally mild and transient	Palatability (taste); Daily dosing frequency; Storage conditions	Araujo and Botelho (2022), Liu L. et al. (2023), Deng et al. (2024), Terren Lora et al. (2024), He et al. (2025)
Prebiotics	Bloating, borborygmi (stomach rumbling), increased flatulence, mild abdominal pain	Similar to placebo Symptoms are typically dose-dependent (fermentation-related) but do not differ significantly from control groups in aggregate analyses	Dosage titration required (start low, go slow); Concurrent fluid intake	Erhardt et al. (2023), Deng et al. (2024), Erhardt et al. (2024), Terren Lora et al. (2024)
Synbiotics	Combined symptoms of above (bloating, mild diarrhea), varying by formulation	Good tolerability No serious adverse events attributed to treatment. Overall adverse event rates show no statistical disparity compared to placebo	Patient education on initial transient symptoms to prevent dropout	Liu L. et al. (2023), Deng et al. (2024), Terren Lora et al. (2024)

AEs, Adverse Events. Sources: Data derived from the safety analyses in the systematic reviews and RCTs, included in this review (e.g., Deng et al., 2024; Liu L. et al., 2023; Terren Lora et al., 2024).

### 6.2 Regulatory status and clinical risk management

The regulatory framework for PP/S varies significantly by region. In the United States, the FDA distinguishes between dietary supplements and “Live Biotherapeutic Products” (LBPs) regulated as drugs; the European Union enforces strict health claim substantiation under EFSA; and China operates a dual-track system involving “Health Food” (SAMR) and pharmaceutical (NMPA) pathways (Cordaillat-Simmons et al., 2020). Despite these evolving frameworks, currently, in many jurisdictions, these products are still regulated as dietary supplements or foods rather than pharmaceuticals. This classification often implies less stringent pre-market approval requirements compared to drugs, potentially leading to variability in product potency and purity. Moving forward, the development of LBPs intended for specific medical indications (such as treating FC-related depression) will require adherence to stricter pharmaceutical standards regarding strain characterization, stability, and manufacturing quality (GMP) (Jin et al., 2024; Zhang et al., 2025). From a clinical risk management perspective, ensuring strain traceability and batch-to-batch consistency is paramount to guarantee reproducible therapeutic effects. Clinicians must exercise heightened vigilance, strictly assessing contraindications to avoid use in high-risk immunocompromised populations. Furthermore, future RCTs are urged to implement systematic pharmacovigilance protocols to rigorously capture and report adverse events, moving beyond the passive monitoring often seen in nutritional studies.

## 7 Discussion

### 7.1 Summary of main findings

This review synthesizes the current evidence on the use of PP/S for managing functional constipation (FC) and its co-occurring depressive symptoms. Data suggest that PP/S interventions are generally effective in increasing stool frequency in adults, with synbiotics showing promising efficacy (Deng et al., 2024). However, evidence in pediatric populations remains inconsistent. Regarding psychological outcomes, while “psychobiotics” have demonstrated efficacy in primary depression, evidence for their specific benefit in “FC-related depression” remains preliminary and largely extrapolated. As summarized in Table 3, trials evaluating dual outcomes [e.g., (Zhang X. et al., 2021)] often observe improvements in bowel function but non-significant differences in mood scores compared to placebo, highlighting the complexity of gut-brain interactions.

### 7.2 Limitations of existing evidence

Several limitations in the current literature must be acknowledged. First, substantial heterogeneity in study protocols constitutes a primary barrier. Variations in probiotic strains (single vs. multi-strain), dosages (ranging from 10^8^ to 10^11^ CFU), and treatment durations complicate direct cross-study comparisons and the identification of a “gold standard” regimen. Furthermore, there is a distinct scarcity of “dual-targeted” RCTs; most included studies focus singly on either GI symptoms or mood outcomes, with few high-quality trials specifically recruiting patients who meet diagnostic criteria for both functional constipation and depression.

Additionally, the assessment of “dual efficacy” is confounded by the substantial placebo response characteristic of both functional gastrointestinal disorders (FGIDs) and mild-to-moderate depression. In many trials, symptom improvements in the placebo group nearly match those in the intervention group, making it difficult to isolate the specific biological effect of the probiotic. Consequently, much of the evidence supporting dual benefits remains exploratory rather than definitive (Zhang X. et al., 2021).

Crucially, a distinction must be drawn between statistical significance and clinical relevance. While several studies reported statistically significant improvements, few explicitly interpreted these findings in the context of Minimal Clinically Important Differences (MCIDs). For constipation, an increase of ≥1 CSBM/week is commonly used as a clinically meaningful threshold in regulatory guidance (U.S. Food and Drug Administration, 2012). Similarly, for depression scores (e.g., HAMD), a reduction of ≥3 points is generally required to reflect a perceptible alleviation of symptoms (Leucht et al., 2013); thus, statistically significant reductions of smaller magnitude (e.g., 1–2 points) may lack tangible clinical impact. The current scarcity of MCID-based responder rate reporting represents a critical gap, and future trials should prioritize these established thresholds to validate real-world therapeutic value.

Methodologically, as a narrative review, this study provides a qualitative synthesis but does not perform a quantitative meta-analysis. Unlike systematic reviews, this review did not involve a formal risk of bias (RoB) assessment or quantitative quality grading (e.g., GRADE) for all included studies. Consequently, potential selection bias cannot be entirely ruled out, and the strength of the synthesized evidence should be interpreted with appropriate caution.

### 7.3 Future directions

Future research should prioritize: (1) Dual-Targeted RCTs specifically recruiting FC patients with comorbid mood disorders using Rome IV and DSM-5 criteria; (2) Mechanistic Biomarkers incorporating multi-omics to identify specific metabolites correlating with clinical gains; and (3) Standardization of PP/S formulations to enable meaningful comparisons. Furthermore, future research should compare functional constipation with constipation secondary to neurological disorders [e.g., Parkinson’s disease (Ghalandari et al., 2023)] to better isolate specific gut-brain axis pathways.

## 8 Conclusion

Current evidence suggests that in adults, including some elderly individuals, probiotics and prebiotics are associated with improvements in stool frequency and consistency while alleviating constipation-related symptoms. These benefits are relatively stable across multiple RCTs, although moderate heterogeneity persists regarding specific products (Mitelmao et al., 2021; Sola et al., 2022; Wallace et al., 2022; Recharla et al., 2023; Deng et al., 2024). In distinct contrast, evidence in children remains inconsistent or non-significant, indicating that efficacy data from adults cannot be simply extrapolated to pediatric populations (Wallace et al., 2022; Dong et al., 2023; Liu X. et al., 2023; Zhang Y. et al., 2023).

Regarding depressive outcomes, while psychobiotic trials in primary depression suggest small-to-moderate improvements (approximately 2–3.5-point reductions), evidence for such “dual efficacy” specifically in FC cohorts remains preliminary, largely extrapolated, and potentially confounded by the high placebo response characteristic of functional disorders; mood outcomes are mostly secondary/exploratory. As persistence beyond 6 months requires further confirmation and direct evidence in FC is limited, these findings suggest that probiotics and prebiotics are currently most suitable as adjunctive rather than replacement therapies (El Dib et al., 2021; Gawlik-Kotelnicka et al., 2023; Zhang Q. et al., 2023; Asad et al., 2025; Soderberg Veiback et al., 2025).

Overall safety and tolerability are favorable; as detailed in Table 4, adverse events were primarily mild, transient gastrointestinal discomfort, with no significant increase in risk compared to placebo (Araujo and Botelho, 2022; Terren Lora et al., 2024). While synbiotic or multi-strain regimens show potential, limitations in strain selection, dosage, and standardization remain. Consequently, large-scale, multicenter, dual-targeted RCTs with standardized protocols are urgently needed to confirm whether these extrapolated benefits translate to clinical populations with comorbid FC and depression. To guide clinical practice and future research, we provide a stratified summary of evidence quality and clinical implications (see Table 5), along with specific principles for standardized study design (see Table 6).

**TABLE 5:** Stratified summary of evidence quality and clinical implications by population and outcome domain.

Population	Intervention	Key findings: GI outcomes	Key findings: psychological outcomes	Strength of evidence and clinical implication	References
Adults	Probiotics (e.g., Bifidobacterium, *Lactobacillus*)	Effective. Consistent improvements in stool frequency (SBM/CSBM) and consistency (BSFS) reported in multiple RCTs	Preliminary/mixed. Statistically significant reduction in depression scores often observed, but “dual efficacy” in FC-specific cohorts is not yet definitive	Moderate. Recommended as adjunctive therapy. High heterogeneity in strains and dosages remains a limitation	El Dib et al. (2021), Mitelmao et al. (2021), Zhang X. et al. (2021)
Adults	Prebiotics/synbiotics	Effective. Improvements in colonic transit time and stool frequency; Synbiotics may show synergistic effects	Limited data. Some positive signals in mood regulation via SCFA production, but fewer dedicated RCTs compared to probiotics	Low to Moderate. Potential alternative, but optimal combinations require further standardization	Recharla et al. (2023), Deng et al. (2024)
Elderly	Probiotics/synbiotics	Likely effective. Improvements in constipation symptoms appear similar to the general adult population	Insufficient data. Very few trials specifically assess mood outcomes in the elderly with FC.	Low. Use with caution; efficacy extrapolated largely from general adult studies	Sola et al. (2022), Asad et al. (2025)
Pediatrics	Probiotics/synbiotics	Inconsistent/no benefit. Meta-analyses indicate no significant improvement in treatment success or stool frequency compared to placebo	Insufficient data. Almost no high-quality evidence evaluating psychological outcomes of PP/S in children with FC.	Insufficient. Not routinely recommended due to lack of efficacy and high placebo response rates in children	Wallace et al. (2022), Dong et al. (2023), Liu X. et al. (2023)

Abbreviations: FC, functional constipation; GI, gastrointestinal; SBM, spontaneous bowel movement; CSBM, complete spontaneous bowel movement; BSFS, Bristol Stool Form Scale; SCFA, short-chain fatty acids; PP/S, probiotics, prebiotics, and synbiotics; RCT, randomized controlled trial.

Strength of Evidence: The ratings (Moderate, Low, Insufficient) represent a qualitative synthesis of the consistency and quality of included studies, rather than a formal quantitative grading (e.g., GRADE) assessment.

Summary of Clinical Relevance: In adults, PP/S are associated with improvements in GI outcomes that often exceed a ≥1 bowel movement/week clinical anchor; however, CSBM-based MCID (≥1 CSBM/week) cannot be consistently judged because CSBM and MCID-based responder rates are not uniformly reported. In contrast, psychological improvements are generally small and heterogeneous; when HAMD is reported, reductions frequently fall below the MCID of 3 points, and mood outcomes are often secondary/exploratory, so these signals should be interpreted as preliminary.

**TABLE 6:** Stratified benefit and study design recommendations comparison table.

Stratification factor	Potentially more beneficial populations/Scenarios	Design and implementation recommendations	References
Baseline fiber/diet	Individuals with low fiber intake	Dietary recording, co-intervention restriction	Araujo and Botelho (2022), Deng et al. (2024), Terren Lora et al. (2024)
Comorbidities/psychological characteristics	IBS-C/anxiety co-occurrence	Preset subgroups, combined scales	Recharla et al. (2023), Shatri et al. (2023), Zhang Q. et al. (2023)
Concurrent medication use	Laxative/antidepressant users	Set combined/sequential arms	Araujo and Botelho (2022), Yang et al. (2022), Terren Lora et al. (2024)
Endpoint definition	Unified SBM/CSBM, BSFS, MCID	Primary + key secondary endpoints	Zhang Q. et al. (2023), Deng et al. (2024), Zandifar et al. (2025)
Follow-up duration	High risk of maintenance/relapse	Follow-up ≥6–12 months	Zhang Q. et al. (2023), Terren Lora et al. (2024), He et al. (2025)

Abbreviations: IBS-C, Irritable Bowel Syndrome with Constipation; SBM, spontaneous bowel movement; CSBM, complete spontaneous bowel movement; BSFS, Bristol Stool Form Scale; MCID, Minimal Clinically Important Difference.

Clinical Relevance: MCID generally refers to a clinically meaningful improvement, such as an increase of ≥1 CSBM/week for constipation outcomes.

## Glossary

5-HT: 5-hydroxytryptamine (serotonin)
AEs: adverse events
BDI: Beck Depression Inventory
BDNF: brain-derived neurotrophic factor
BSFS: Bristol Stool Form Scale
CFU: colony-forming units
CI: confidence interval
CRP: C-reactive protein
CSBM: complete spontaneous bowel movements
DSM-5: Diagnostic and Statistical Manual of Mental Disorders, Fifth Edition
FC: functional constipation
FOS: fructo-oligosaccharides
GABA: gamma-aminobutyric acid
GI: gastrointestinal
GMP: Good Manufacturing Practice
GOS: galacto-oligosaccharides
HADS: Hospital Anxiety and Depression Scale
HAMD: Hamilton Depression Rating Scale
IL-1β: interleukin-1 beta
IL-6: interleukin-6
LBPs: live biotherapeutic products
MCID: Minimal Clinically Important Difference
MD: mean difference
MeSH: Medical Subject Headings
MGBA: microbiota–gut–brain axis
Mix7: synbiotic mixture “Mix7” (as named in the cited analysis)
PP/S: probiotics, prebiotics, and synbiotics
PRISMA: Preferred Reporting Items for Systematic Reviews and Meta-Analyses
RCT: randomized controlled trial
RCTs: randomized controlled trials
Rome III: Rome III diagnostic criteria
Rome IV: Rome IV diagnostic criteria
SBM: spontaneous bowel movements
SCFA: short-chain fatty acid
SCFAs: short-chain fatty acids
SMD: standardized mean difference
SUCRA: surface under the cumulative ranking curve
TNF-α: tumor necrosis factor-alpha
